# Efficiency and Individual Freedom

**Published:** 1917-09-29

**Authors:** 


### The Hospital

The Workers' Newspaper of Administrative Medicine and Institutional Life, Administration, National Insurance and Health.

No. 1634, Vol. LXII. SATURDAY, SEPTEMBER 29, 1917. PRICE ONE PENNY

### EFFICIENCY AND INDIVIDUAL FREEDOM.

The stress and strain of a great war constitute a testing-time both for men and for principles, and under their influence the standards of judgment inevitably become readjusted. The trial involves issues of such tremendous and immediate importance that public opinion is apt to see these to the exclusion of all others. When the existence of the country is at stake, not to speak of any question of personal peril or loss, everything which will avert the disaster comes necessarily into the first rank, and other considerations fall into the background. The verdict is forced by the imminence and greatness of the claim, and the new conditions dominate the immediate horizon. They do even more than this. While all allow that there is a future which is to be written in terms of peace, judgment on its problems is influenced by the tests which have been born in the hour of war. The qualities which seem eminent and effective in the emergency appear to command the whole situation and are exalted as the most worthy guides to the whole scheme of national life. There is no need seriously to quarrel with this tendency, at least at the moment, seeing that it helps to arouse and to maintain the energy and endurance which the occasion essentially requires. Obviously the first claim is to preserve national existence, while the fixing of its conditions may be postponed to a more convenient season. Yet it is not unreasonable to remember that peace and war are states which present many oppositions, and that it is not a secure conclusion to reason that what may be excellent and even necessary in the one has equal virtues when applied to the other. The confusion which leads to such a verdict may be excused so long as it gives help and stimulus in the emergency, but to accept it as logically justified means a structure resting on a premature, and possibly on an erroneous, foundation. Analogies have a taking quality, and they readily appeal to an interested audience, but reflection often discovers defects which are fatal to the whole of the argument. In the present instance it may be wise to vaunt the qualities which make successful war, for these at the moment are vital to the issue which is in debate. But so politic a procedure ought not to mean that these qualities must also hold the first place when peace is restored and the life of the nation is re-established in its natural channel. What will help here and now must indeed have its opportunity, but the time must come when it will have to be appraised under new conditions. That the judgment will be the same then as now may to some appear probable. But to accept such a conclusion as certain is to forget that the morrow will have its own circumstances, and that the virtues of to-day may, in view of these, pass into a different rank from the one which they now occupy.

At the moment, very naturally and properly, organisation, efficiency, and obedience receive unstinted admiration, for it is on the effects which go out from these qualities that the national cause depends. Persons who set themselves in opposition to such demands come under general condemnation as wilful, stupid, wrong-headed, and unpatriotic. The time is not one for individual assertion but for individual repression, and law and order and regulation are the necessities of the hour. Only by their provision and enforcement can the will of the nation see its triumph within sight. Hence the spirit which underlies them and the ends to which they lead are in the existing circumstances recognised as having supreme value, and whether they possess or do not possess attendant disadvantages is a question which does not arise. Or rather, if we judge by some utterances, such a question is already answered in the negative. Efficiency and organisation and obedience are at the top of the scale. They have been justified by one great scheme of national politics, and if our opposing enterprise has now achieved some measure of success it is because we have copied, and perhaps bettered, our enemy's teaching. Hence the debate is closed for all time, and the obvious lesson is that the same principles which are serving us at the moment must be permanently adopted as the guiding forces of the life of the nation. At the moment it may seem almost treasonable to hint a doubt of the value of this conclusion. Nevertheless, doubts are likely to arise. Events which to-day are passed without notice, because of the acute and commanding claim which arrests all our attention, will if repeated in times of peace compel acute feeling and even strong opposition. Governing authorities at present find a smooth path because their orders are regarded as essential to an aim greatly desired—namely, a triumph over our enemies. And men consent to restrictions and to compulsion because opposition would seem inconsistent with this aim. But with a new day older habits and manners of thought are likely to reassert themselves, and while certain of the powers that be may naturally desire to continue to govern us for our good there will be minds who will make it their business to see that we are not governed overmuch. Principles such as are here presented are in operation, and are likely to be in operation, in the world of medicine. The order and organisation of the moment exercise their attraction, and ambitions are cultivated to extend these virtues into the time of peace. Ruling powers, doubtless with the best of motives, hope to enlarge and to enforce their control and to impose wise conduct by the force of law. At present fortune appears to smile upon them. But the time of testing has yet to come, and it may, perhaps, then be discovered that neither practitioners nor patients will bear patiently official chains. The schemes are admirable, but they carry as a penalty some sacrifice of individual freedom, and, sad as it may seem, there may be those who will conclude that even efficiency may be paid for with too high a price.

